# Early diagnosis of sepsis-associated AKI: based on destruction-replenishment contrast-enhanced ultrasonography

**DOI:** 10.3389/fmed.2025.1563153

**Published:** 2025-03-25

**Authors:** Zexing Yu, Xue Shi, Yang Song, Xin Li, Ling Li, Huiyu Ge

**Affiliations:** Department of Ultrasound Medicine, Beijing Chaoyang Hospital, Capital Medical University, Beijing, China

**Keywords:** destruction-replenishment contrast-enhanced ultrasound, deep learning ultrasound radiomics model, acute kidney injury, risk assessment, deep learning model

## Abstract

**Objective:**

Establish a deep learning ultrasound radiomics model based on destruction-replenishment contrast-enhanced ultrasound (DR-CEUS) for the early prediction of acute kidney injury (SA-AKI).

**Method:**

This paper proposes a deep learning ultrasound radiomics model (DLUR). Deep learning models were separately established using ResNet18, ResNet50, ResNext18, and ResNext50 networks. Based on the features extracted from the fully connected layers of the optimal model, a deep learning ultrasound radiomics model (DLUR) was established using three classification models (built with 3 classifiers). The predictive performance of the best DLUR model was compared with the visual assessments of two groups of ultrasound physicians with varying levels of experience. The performance of each model and the ultrasound physicians was evaluated by assessing the receiver operating characteristic (ROC) curves. The area under the curve (AUC), sensitivity, specificity, positive predictive value (PPV), negative predictive value (NPV), and accuracy were subsequently calculated.

**Results:**

Compared to the ResNet18 model, the DLUR model based on logistic regression (DLUR-LR) demonstrated the best predictive performance, showing a Net Reclassification Improvement (NRI) value of 0.210 (*p* < 0.05). The Integrated Discrimination Improvement (IDI) value for the corresponding stage was 0.169 (*p* < 0.05). Additionally, the performance of the DLUR-LR model also surpassed that of senior ultrasound physicians (AUC, 0.921 vs. 0.829, *p* < 0.05).

**Conclusion:**

By combining deep learning and ultrasound radiomics, a deep learning ultrasound radiomics model with outstanding predictive efficiency and robustness has demonstrated excellent capability in the early prediction of acute kidney injury (SA-AKI).

## 1 Introduction

According to the 2020 WHO statistics (1), there were 48.9 million cases of sepsis worldwide in 2017, resulting in 11 million deaths. Sepsis-related deaths accounted for 19.7% of all global deaths. The mortality rate of sepsis is 15–25%, and this rate increases to 30–50% in cases of septic shock. Therefore, sepsis represents a significant public health issue worldwide due to its high incidence and mortality rates.

The kidneys are one of the organs most frequently affected by sepsis. Poston and Koyner (2) pointed out that up to 60% of sepsis patients develop secondary AKI, and the mortality rate significantly increases once sepsis is complicated by AKI. It is currently believed (3) that sepsis triggers macrocirculatory disturbances, leading to reduced renal blood flow (RBF), which causes acute tubular necrosis, thereby resulting in sepsis-associated AKI. As research into sepsis-related AKI deepens, studies (4, 5) have found that during septic shock, despite maintained or even increased RBF, the glomerular filtration rate (GFR) decreases, suggesting that the pathogenesis of sepsis-related AKI may be more complex. Hence, studying intrarenal blood perfusion has become a crucial step in understanding the pathophysiology of AKI during septic shock.

Currently, there are few methods available to assess and monitor renal cortical microcirculatory perfusion in sepsis patients (6, 7). Conventional color Doppler ultrasound, widely used for real-time monitoring of renal hemodynamics in large vessels and some small vessels in the renal parenchyma (8), lacks accuracy in evaluating microcirculatory perfusion, especially in the renal cortex. The recently developed contrast-enhanced ultrasound (CEUS) technology, which uses microbubble contrast agents (ultrasound contrast agents, UCA) much smaller than red blood cells, allows assessment of human microcirculatory perfusion by reaching any terminal small vessels via the pulmonary circulation.

Concurrently, the rapid advancements in deep learning and artificial intelligence have revolutionized medical image analysis, demonstrating exceptional capabilities in feature extraction and pattern recognition (9). Radiomics, which involves the extraction of a large number of quantitative features from medical images, combined with machine learning algorithms, has shown promise in achieving precise disease diagnosis and prognostic predictions (10). However, current radiomics research on SAKI predominantly focuses on modalities such as magnetic resonance imaging (MRI) and computed tomography (CT), with limited studies exploring deep learning-based ultrasound radiomics models for SAKI (11).

To the best of our knowledge, no study has yet confirmed the feasibility of using a DLUR model for the early prediction of acute kidney injury (SA-AKI). This study aims to establish a deep learning ultrasound radiomics model based on burst-replenishment contrast-enhanced ultrasound for the early prediction of acute kidney injury (SA-AKI).

## 2 Materials and methods

### 2.1 Study participants

**Figure 1:**
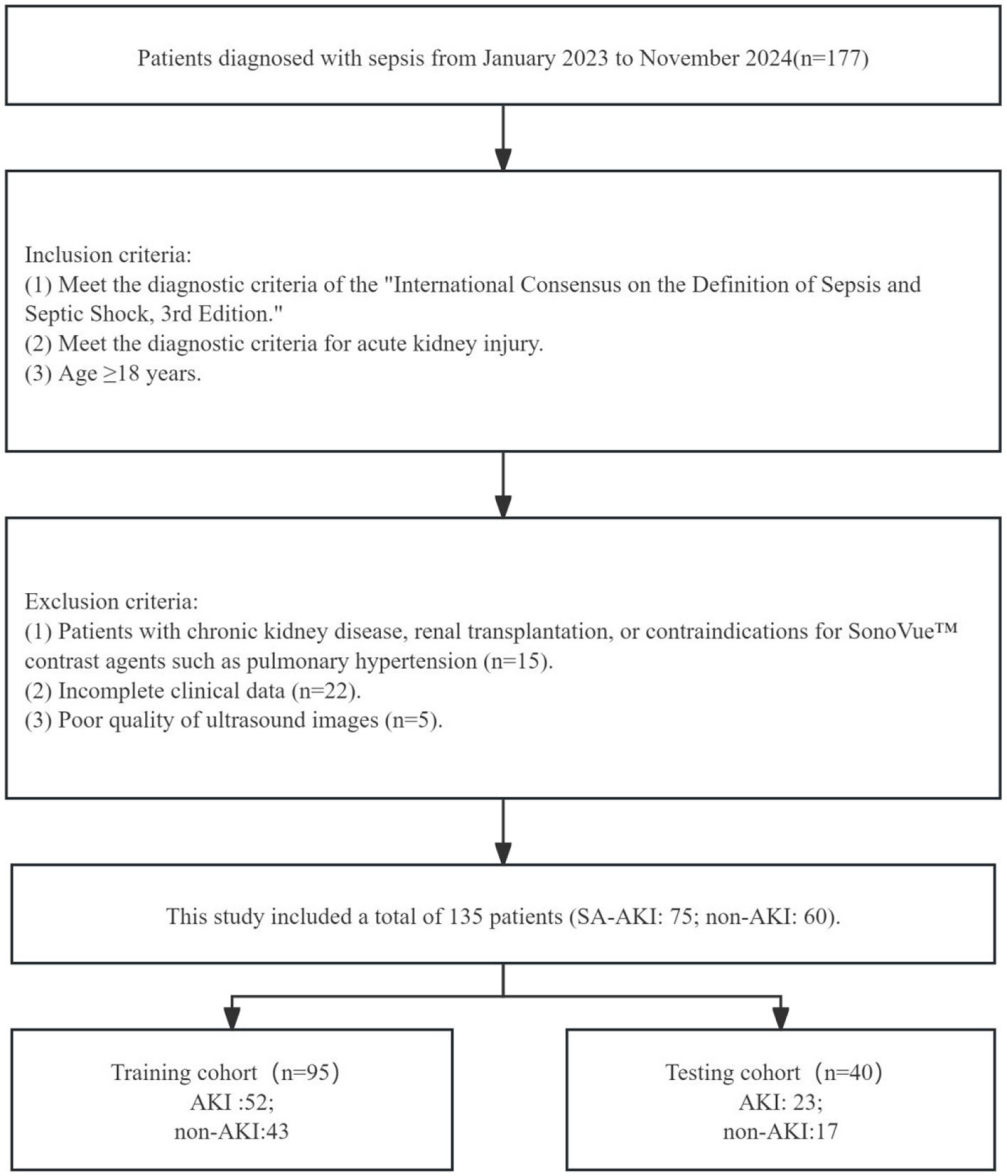
Flow diagram of the study population.

The retrospective study collected data from 135 patients with sepsis at Beijing Chaoyang Hospital, Capital Medical University, from January 2023 to November 2024, including 75 SA-AKI patients and 60 SA-non-AKI patients. The inclusion criteria were: (1) meeting the diagnostic criteria of the “International Consensus on the Definition of Sepsis and Septic Shock, 3rd Edition”; (2) meeting the diagnostic criteria for acute kidney injury: Acute Kidney Injury (AKI) is defined when either of the following criteria is met: (1) Serum Creatinine Elevation Absolute increase in serum creatinine ≥0.3 mg/dL (26.5 μmol/L) within 48 h, OR Serum creatinine rising to ≥1.5 times baseline value (i.e., ≥50% increase from baseline) within 7 days. (2) Urine Output Reduction Sustained urine output < 0.5 mL/kg/h persisting for ≥6 h; (3) age ≥18 years; (4) clear ultrasound images and complete clinical data. The exclusion criteria were: (1) patients with chronic kidney disease, renal transplantation, contraindications for SonoVue™ contrast agents, or pulmonary hypertension; (2) incomplete clinical data; (3) poor quality of ultrasound images. All patients provided informed consent. Please refer to Figure 1 for detailed information. The data from 135 patients with sepsis were randomly divided into a training set (*n* = 95) and a testing set (*n* = 45) in an 7:3 ratio. Input data included burst-reperfusion ultrasound contrast agents and clinical data, while the output indicated whether the patient belonged to the septic AKI or non-AKI group.

### 2.2 Ultrasound data acquisition

Ultrasound diagnosis was performed by physicians with more than 5 years of relevant experience using the Mindray Resona R9 color Doppler ultrasound diagnostic system manufactured by the Chinese medical device company Mindray. The procedure utilized intravenous infusion combined with burst-replenishment contrast-enhanced ultrasound technology. The patient was placed in a supine position, and a vein in the left elbow was punctured to establish an intravenous access using a special vein tube for contrast-enhanced ultrasound. Two vials of SonoVue contrast agent were dissolved in 10 ml of saline, thoroughly shaken, and then placed in a 20 ml syringe. The syringe was installed in a specialized micro-infusion pump for contrast agents, with the speed set at 2 ml/min, and connected to the venous tube. The largest coronal section of the patient's right kidney (showing the renal hilum) was selected for observation. The injection pump was activated, and the ultrasound was used to observe the time it took for the contrast to reach the kidney, followed by continuous observation for 2 min until the contrast entered the kidney parenchyma and reached equilibrium. A fixed high mechanical index (MI > 0.7) was used to continuously burst the microbubbles within the kidney parenchyma for 6 s until all the contrast microbubbles were extinguished. Subsequently, the ultrasound probe was placed at the largest coronal section of the right kidney to continuously and dynamically observe the replenishment phase when the microbubbles re-entered the kidney for 30 s. This burst-replenishment process was repeated three times to obtain three sets of dynamic replenishment images, which were then subjected to time-intensity curve (TIC) analysis to acquire the replenishment data.

### 2.3 Ultrasound image annotation

After anonymizing patient information, the original dynamic ultrasound images were imported into the MedAI Darwin learning platform. The patient information labels were defined as follows: gender, age, body mass index, mean arterial pressure, arterial carbon dioxide partial pressure, hemoglobin, white blood cell count, lactate, and serum creatinine. The lesion information label included: renal function impairment (septic AKI vs. non-AKI). Physicians with over 5 years of relevant experience manually delineated the regions of interest (ROI). In case of discrepancies, consultation with senior physicians (physicians with ≥10 years of ultrasound diagnostic experience and the title of **Associate Chief Physician** or higher) was sought for a definitive diagnosis.

### 2.4 Deep learning ultrasound radiomics model development

To ensure the integrity and validity of the research data, we have undertaken data preprocessing, aiming to enhance the performance and robustness of the models. The data preprocessing steps encompass data augmentation and image normalization. Considering the unique structural characteristics of the training data and the objectives of the task, we chose to build deep learning models based on four different algorithms: ResNet18, ResNet50, ResNeXt18, and ResNeXt50. ResNet (Residual Network) and ResNeXt (Residual NeXt) are highly acclaimed deep learning models in the field of image recognition. They utilize the concept of residual learning, which enables the development of deeper networks without being hindered by issues of vanishing or exploding gradients.

ResNet addresses the issue of degradation in deep convolutional neural networks by introducing residual blocks. In these blocks, the input feature maps are combined with the subsequent layers through skip connections, allowing for the maximum preservation of the original information. Such design enables the residual blocks to learn the residual function, capturing the difference between the feature maps and the desired output. ResNeXt, an improvement upon ResNet, introduces grouped convolution within each residual block to enhance the model's expressive power. Traditional convolutional operations convolve each channel of the input feature maps with each filter, whereas grouped convolution divides the input feature maps into multiple groups and independently convolves each group. By increasing the number of groups, ResNeXt enhances the model's expressive power without increasing the total number of parameters or computational complexity. Typical structures of ResNet and ResNeXt consist of multiple residual blocks, with variants such as ResNet-18, ResNeXt-18, ResNet-50, and ResNeXt-50 being widely used. Both ResNet and ResNeXt are composed of several residual blocks. Within each residual block, the convolutional layers are no longer ordinary convolutions but rather grouped convolutions, which divide the input feature maps into multiple groups for independent convolutional operations. The number of convolutional kernels within each group in grouped convolution is equal, and the quantity of groups is referred to as “cardinality.” By increasing the cardinality, the model's non-linear expressive power can be enhanced. For instance, ResNet-18 is a relatively shallow ResNet model with approximately 11 million total parameters, while ResNet-50 is a deeper and more complex ResNet model with approximately 23 million total parameters.

In general, ResNet and ResNeXt exhibit slight differences in their model structures, but both leverage the concept of residual learning to address challenges in deep networks. These models have demonstrated outstanding performance in image recognition tasks and have become pivotal models in research and applications. The predictive performance of each model is evaluated using receiver operating characteristic (ROC) curves, and metrics such as area under the curve (AUC), sensitivity, specificity, and accuracy are calculated to select the best differentiating model for tuberculous hydronephrosis and non-tuberculous hydronephrosis. To optimize computational resources and improve training efficiency, this study uniformly employs region of interest (ROI) sub-images for model training, with the ROI sub-image size standardized to 64 × 64 × 64 prior to training. Additionally, 3D image augmentation techniques, such as random flipping and random cropping, are applied to the training data.

**Figure 2:**
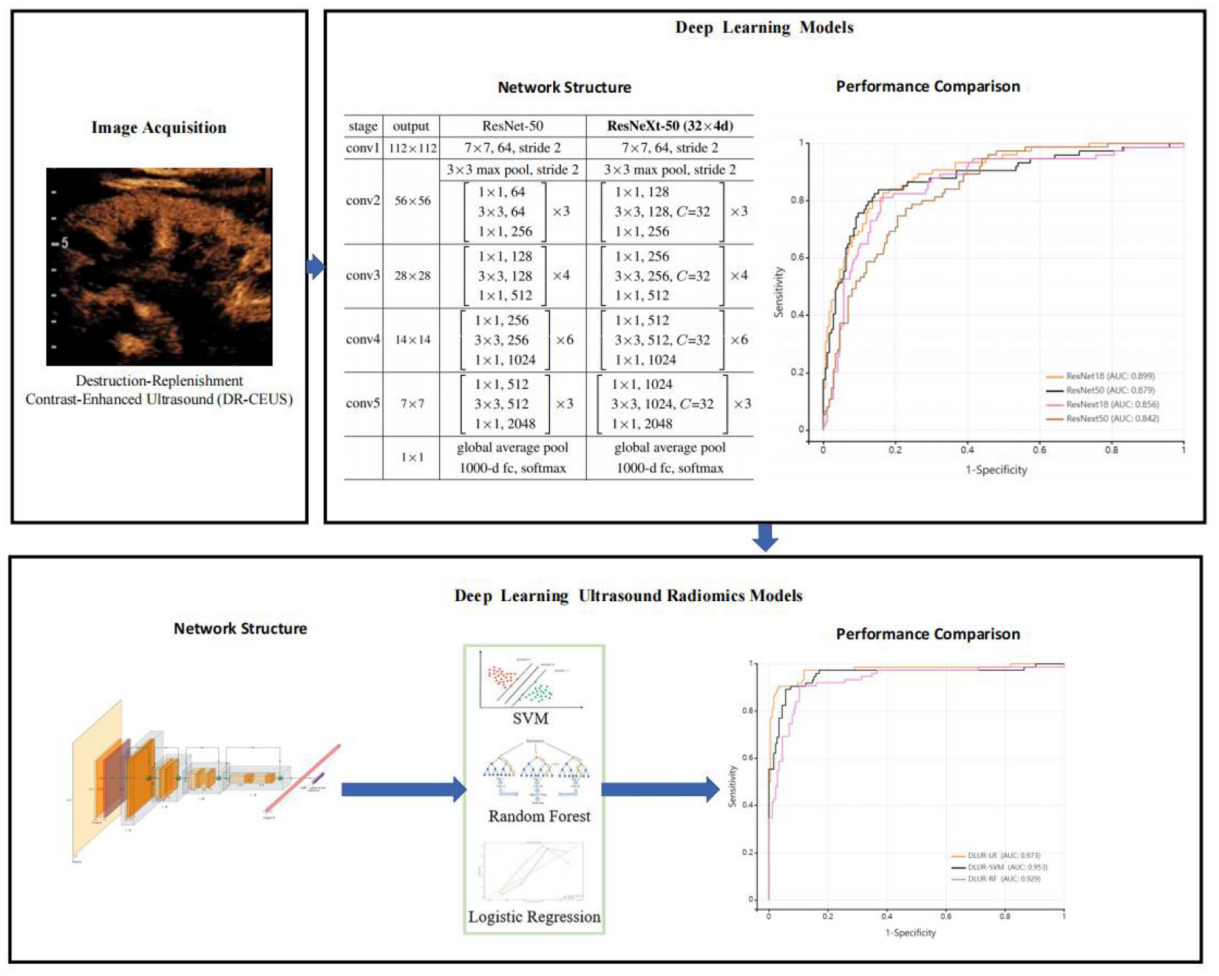
The complete research process.

After evaluating the deep learning modeling experiments, features were extracted from the fully connected layers of the best-performing deep learning model. These deep learning features were then used to build an ultrasound radiomics model using three mainstream machine learning algorithms: Logistic Regression (LR), Support Vector Machine (SVM), and Random Forest (RF). The predictive performance of each model was assessed using receiver operating characteristic (ROC) curves, and metrics such as the area under the curve (AUC), sensitivity, specificity, PPV, NPV, and accuracy were calculated. The complete research process is shown in Figure 2.

### 2.5 Statistical analysis

SPSS version 27.0 statistical analysis software was used to analyze the significance of each model. Categorical data were presented as actual frequencies and percentages. The classification performance of the models was assessed using the AUC, accuracy, sensitivity, specificity, PPV, and NPV derived from the receiver operating characteristic (ROC) curves. The DeLong test was used to compare the significance of the AUCs among the different models. A *P*-value of < 0.05 was considered statistically significant, indicating a difference with practical importance.

## 3 Results

### 3.1 General clinical data

This study included 135 septic patients, who were divided into AKI group (*n* = 75) and non-AKI group (*n* = 60) based on renal function within 48 h and urine output within 24 h. There were 90 males and 45 females, with an average age of 65.3 ± 15.2 years. General clinical data are presented in Table 1. There were no statistically significant differences in age, sex, body mass index, MAP, PaCO2, and CRP between the two groups (*P* > 0.05). The levels of Scr and Lac in the AKI group were significantly higher than those in the non-AKI group, and the differences were statistically significant (*P* < 0.05).The general clinical data of enrolled patients are shown in Table 1.

**Table 1:** The general clinical data of enrolled patients.

	**SA-Non-AKI (*n =* 60)**	**SA-AKI (*n =* 75)**	**Significance (*p*)**
Age	69 (17–90)	63 (54–77)	
Gender (percentage of females)	36%	27%	
Temperature	36.8 ± 0.87	36.8 ± 0.29	0.336
Pulse	82.7 ± 13.2	89.1 ± 21	0.49
Respiration (breaths per minute)	21.4 ± 4.8	18.7 ± 4.7	0.302
BMI	23.8 ± 2.98	22.9 ± 4.9	1.000
Mean arterial pressure (MAP)	72.0 ± 19.0	85.1 ± 9.7	0.193
Arterial partial pressure of carbon dioxide (PaCO2)	54.5 ± 27.5	47 ± 15.8	0.530
Hemoglobin	108.1 ± 24.3	90 ± 22	0.964
White blood cell count	11.7 ± 6.7	15.4 ± 13.3	0.151
Lactic acid	1.26 ± 0.26	2.97 ± 3.15	0.007^*^
ScR	60.6 ± 19.9	216.9 ± 149.5	0.025^*^

^*^Indicates that *P* < 0.05. There is a statistically significant difference between the two groups.

### 3.2 Performance of the deep learning ultrasound radiomics model

**Figure 3:**
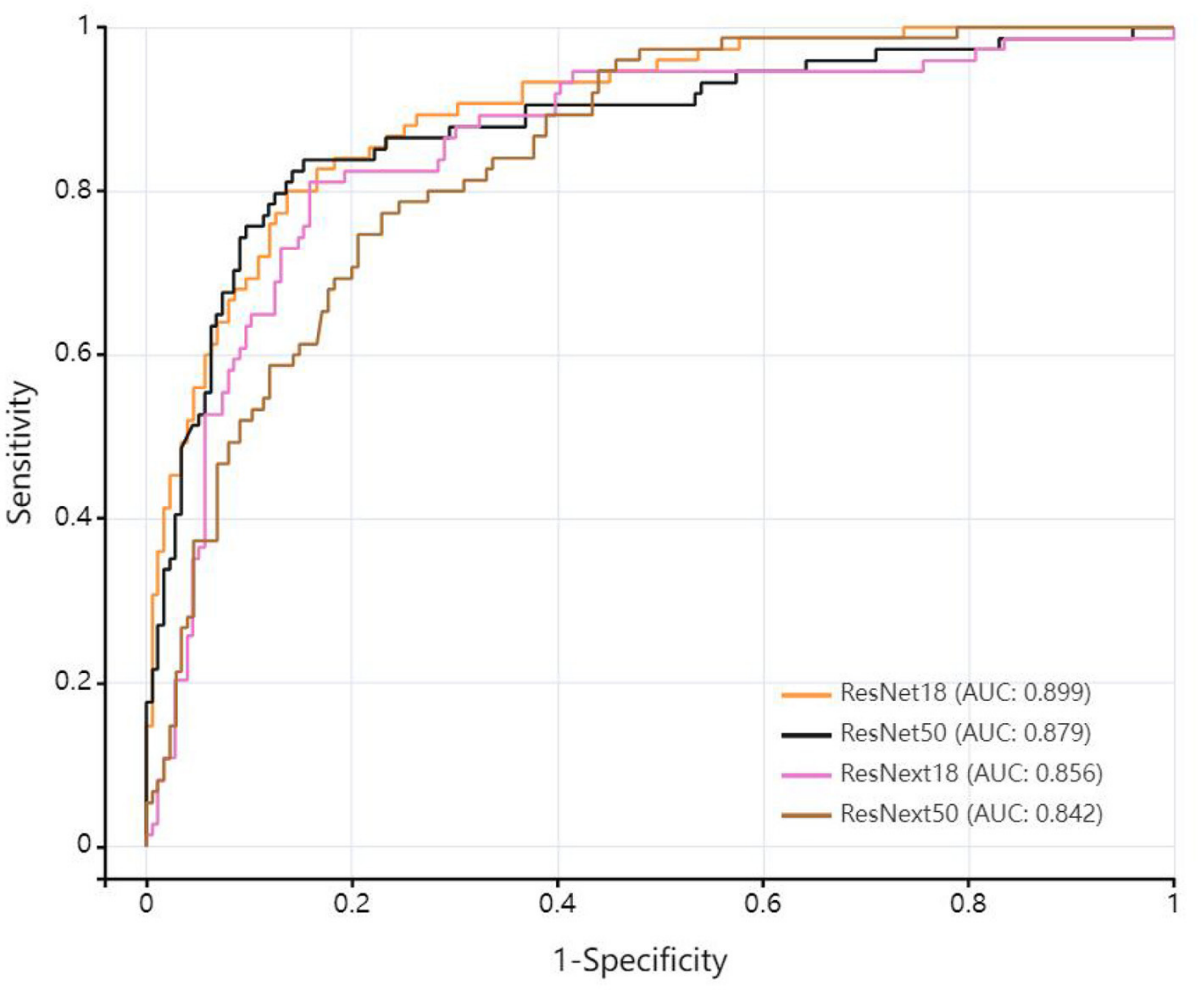
Comparison of performance among different deep learning models.

Table 2 lists four algorithm models based on deep learning technology. Compared with other deep learning models on the testing dataset, ResNet 18 exhibited superior overall performance. The AUC of ResNet 18 was 0.899 (95% CI: 0.858–0.940), with a sensitivity of 0.800, specificity of 0.857, PPV of 0.706, NPV of 0.909, and accuracy of 0.840. Comparison of performance among different deep learning models as shown in Figure 3.

**Table 2:** The performance comparison of different deep learning models.

**Model**	**AUC (95%CI)**	**Sensitivity**	**Specificity**	**PPV**	**NPV**	**Accuracy**
ResNet18	0.899 [0.858–0.940]	0.800	0.857	0.706	0.909	0.840
ResNet50	0.879 [0.828–0.931]	0.838	0.847	0.697	0.925	0.844
ResNext18	0.856 [0.802–0.910]	0.811	0.841	0.682	0.914	0.832
ResNext50	0.842 [0.793–0.892]	0.773	0.766	0.586	0.887	0.768

**Figure 4:**
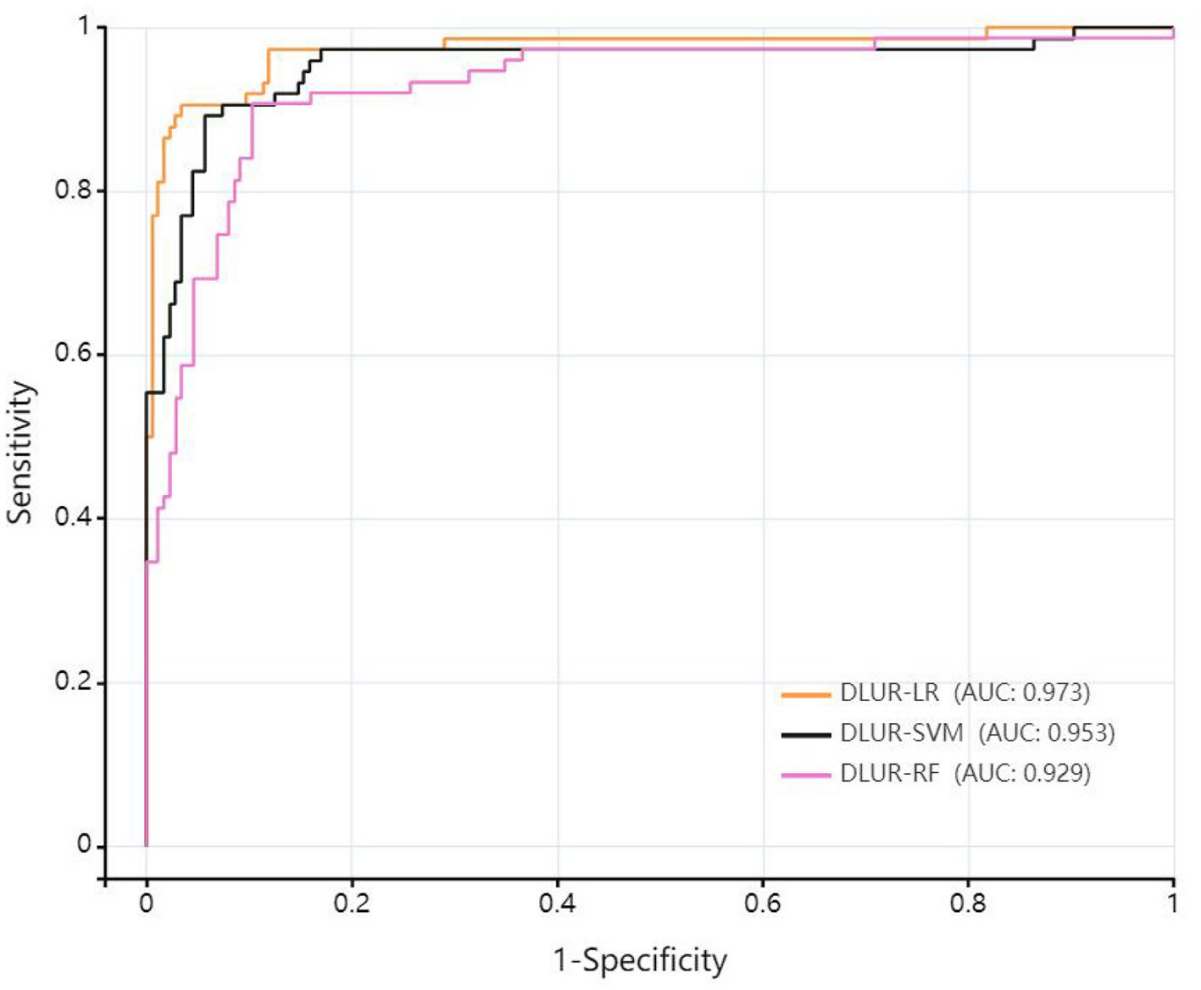
Comparison of performance among different deep learning ultrasound radiomics models.

Ultimately, among the three classifiers, the deep learning ultrasound radiomics model based on logistic regression demonstrated the best classification performance (see Table 3). In the testing set, the AUC of DLUR-LR was 0.973 (95% CI: 0.949–0.998), with a sensitivity of 0.905, specificity of 0.960, PPV of 0.905, NPV of 0.960, and accuracy of 0.944; the AUC of DLUR-SVM was 0.953 (95% CI: 0.918–0.988), with a sensitivity of 0.892, specificity of 0.938, PPV of 0.857, NPV of 0.954, and accuracy of 0.924; the AUC of DLUR-RF was 0.929 (95% CI: 0.890–0.968), with a sensitivity of 0.907, specificity of 0.891, PPV of 0.782, NPV of 0.957, and accuracy of 0.896. Comparison of performance among different deep learning ultrasound radiomics models as shown in Figure 4.

**Table 3:** The performance comparison of different deep learning ultrasound radiomics models.

**Model**	**AUC (95%CI)**	**Sensitivity**	**Specificity**	**PPV**	**NPV**	**Accuracy**
DLUR-LR	0.973 [0.949–0.998]	0.905	0.960	0.905	0.960	0.944
DLUR-SVM	0.953 [0.918–0.988]	0.892	0.938	0.857	0.954	0.924
DLUR-RF	0.929 [0.890–0.968]	0.907	0.891	0.782	0.957	0.896

In our study, we utilized performance metrics such as AUC, sensitivity, specificity, PPV, and NPV due to their significant clinical importance in the context of early AKI diagnosis. These metrics were carefully chosen to align with and reflect the critical aspects of clinical outcomes. The AUC provides a comprehensive assessment of the model's overall ability to distinguish between AKI and non-AKI cases across all thresholds, offering a holistic evaluation of performance. Sensitivity and specificity are directly related to clinical priorities: high sensitivity minimizes missed true cases, ensuring timely treatment, while high specificity reduces false positives, avoiding unnecessary interventions. PPV and NPV further aid clinical decision-making by indicating the likelihood that test results accurately reflect the patient's condition, thereby supporting clinicians in making informed treatment choices. Together, these metrics not only validate the statistical performance of our model but also underscore its practical utility in improving patient outcomes by facilitating early and accurate diagnosis of sepsis-associated AKI.

## 4 Discussion

Sepsis-associated acute kidney injury (AKI) is a significant complication that complicates the management of septic patients and dramatically increases morbidity and mortality rates. The early diagnosis of sepsis-associated AKI is crucial for implementing timely therapeutic strategies, which can significantly improve patient outcomes. In this study, we explored the utility of destruction-replenishment contrast-enhanced ultrasonography (DR-CEUS) as a novel method for the early detection of sepsis-associated AKI.

Our results indicate that DR-CEUS can detect early renal changes associated with sepsis before traditional markers show significant alterations. This is particularly important in the context of sepsis, where timely intervention is necessary to mitigate kidney injury. The ability to identify renal microcirculatory dysfunction may allow clinicians to initiate protective strategies earlier in the disease course, potentially reversing or preventing AKI progression.

We developed a deep learning ultrasound radiomics model that outperforms four different deep learning network models, namely ResNet18, ResNet50, ResNext18, and ResNext50. Compared to the best-performing model within ResNet18, our deep learning ultrasound radiomics model demonstrated superior predictive performance on the explosive-replenishment contrast-enhanced ultrasound imaging test data. The deep learning informatics model exhibited higher reliability and reproducibility in evaluating diagnostic outcomes, leveraging its inherent characteristics.

The performance differences among the various deep learning network models may be attributed to their distinct network architectures (12). In our study, we trained four different deep learning network architectures: ResNet18, ResNext18, ResNet50, and ResNext50, all of which are widely used in various clinical applications. We chose ResNet18, which exhibited the best predictive performance in our study, to extract deep learning features for constructing the deep learning ultrasound radiomics model. Among these four models, the ResNet network demonstrated more stable and superior predictive performance compared to other classical deep learning networks in the test set. The ResNet architecture maintains the integrity of information by directly passing input information to the output to learn the residual functions throughout the network. This property helps mitigate the issues of gradient vanishing and explosion, allowing the network to deepen without compromising performance (13). The ResNext network is a new architecture based on ResNet that incorporates the recurrent layer strategy of ResNet and combines it with a split-transform-merge strategy in a simple and scalable manner (14). However, the predictive results of the ResNext network were inferior to those of the ResNet network. In deep learning, dimensionality reduction, classification, and feature extraction are performed in an integrated manner. However, the quality and output of these cascading layers depend on various hyperparameters such as the number of layers, feature maps, layer configurations, and structures. Different network architectures utilize different sets of hyperparameters, and the choice of these hyperparameters and architectures may impact predictive performance.

Our study reveals that the deep learning ultrasound radiomics model significantly outperforms traditional diagnostic methods and physician assessments in diagnosing sepsis-associated AKI. This advancement holds promise for improving early diagnosis in clinical settings. The integration of such AI-powered tools is increasingly feasible due to advancements in digital healthcare infrastructure, and our model can be seamlessly incorporated into existing ultrasound practices. However, potential barriers include initial investment costs, resistance to workflow changes, a need for comprehensive training, regulatory hurdles, and data privacy concerns. Despite these challenges, the clinical impact of implementing this model is substantial, offering more accurate diagnoses, timely interventions, and improved patient outcomes, while also alleviating physicians' cognitive load. Expanding our discussion to include these integration considerations and clinical benefits highlights the model's potential to enhance real-world healthcare delivery.

## 5 Conclusion

In this study, we propose a deep learning ultrasound imaging model based on blast-reperfusion ultrasound contrast imaging. Our method effectively integrates the technical advantages of deep learning and ultrasound imagingomics, demonstrating excellent predictive performance for the early diagnosis of sepsis-related acute kidney injury (AKI). This enables clinicians to detect renal changes earlier than traditional methods, allowing for the use of more precise interventions.

## Data Availability

The datasets presented in this article are not readily available because the dataset is subject to several restrictions, including limited access, which may require specific permissions or credentials for use. Usage of the dataset is prohibited for commercial purposes without prior authorization, and users must comply with data privacy regulations governing any personally identifiable information (PII) it contains. Additionally, proper attribution is required when the data is cited or referenced in research, and modifications to the dataset are not allowed unless explicitly permitted. Users must carefully review these conditions to ensure compliance with all applicable legal and ethical guidelines. Requests to access the datasets should be directed to yu_zexing@126.com.
